# Serial TAPSE and Subsequent 60-Day Mortality Among Critically Ill Adults with Reduced Baseline TAPSE: A MIMIC-IV-ECHO Cohort Study

**DOI:** 10.3390/jcm15197753

**Published:** 2026-10-08

**Authors:** Shanshan Zhai, Dezhi Ren, Yongyi Chen, Hui Wang, Jun Duan

**Affiliations:** Department of Intensive Care Unit, China-Japan Friendship Hospital, Beijing 100029, China; zhaishanshan@cjfh.org.cn (S.Z.); 18724234308@163.com (D.R.); chenyongyi@cjfh.org.cn (Y.C.); wanghui@cjfh.org.cn (H.W.)

**Keywords:** critical illness, echocardiography, TAPSE, serial assessment, mortality, MIMIC-IV-ECHO

## Abstract

**Background/Objectives:** Tricuspid annular plane systolic excursion (TAPSE) is routinely available in critical-care echocardiography, but the prognostic relevance of serial change is uncertain. We examined serial TAPSE and subsequent mortality among critically ill adults with reduced baseline TAPSE. **Methods:** This retrospective MIMIC-IV-ECHO cohort included adults with at least two TAPSE measurements and baseline TAPSE < 1.7 cm. Serial TAPSE status was classified as improved (increase >0.2 cm) or non-improved (change ≤0.2 cm). Cox regression evaluated 60-day mortality from the follow-up echocardiogram. **Results:** Among 227 patients, 101 (44.5%) were improved and 126 (55.5%) non-improved over a median interval of 10 days. Non-improved status was associated with higher subsequent mortality (adjusted hazard ratio 1.72, 95% confidence interval 1.06–2.80; *p* = 0.028). After adjustment for follow-up TAPSE, neither binary status nor continuous change was significantly associated with mortality. **Conclusions:** In this exploratory study, non-improved serial TAPSE was associated with higher 60-day mortality. Added prognostic value beyond follow-up TAPSE was not demonstrated.

## 1. Introduction

Right ventricular (RV) dysfunction is common in the intensive care unit (ICU) and occurs across a broad spectrum of critical illness, including acute respiratory distress syndrome (ARDS), pulmonary embolism, sepsis, and acute decompensated heart failure [1,2,3,4,5,6]. Acute increases in pulmonary vascular resistance and RV afterload can contribute to RV dysfunction, which may improve as the underlying illness and loading conditions change [2,7]. Monitoring RV function over time may therefore help clinicians assess the course of critical illness.

A recent meta-analysis in patients with sepsis associated RV dysfunction with higher mortality (relative risk 1.62, 95% confidence interval 1.35–1.95) [8]. Echocardiographic assessment includes tricuspid annular plane systolic excursion (TAPSE), RV fractional area change, and the RV/left ventricular (LV) diameter ratio [9,10,11]. TAPSE is simple to obtain and provides a measure of RV longitudinal function, making it practical for repeated bedside assessment [9,10,11]. A single examination, however, cannot capture changes during the course of illness. In COVID-19 ARDS, Huang et al. found that persistent acute cor pulmonale was associated with poorer survival and that acute cor pulmonale on the last examination carried the highest mortality risk [3]. Their study examined changes between categorical RV phenotypes in a single disease, rather than within-patient TAPSE change across different causes of critical illness.

The prognostic relevance of serial TAPSE change beyond baseline severity in a broader critically ill population remains unclear. MIMIC-IV-ECHO provides structured echocardiographic data linked to detailed critical-care records [12,13]. We examined whether binary serial TAPSE status was associated with 60-day mortality after follow-up echocardiography among critically ill adults with reduced baseline TAPSE.

## 2. Materials and Methods

### 2.1. Data Source and Study Design

This retrospective cohort study used MIMIC-IV version 3.1 and MIMIC-IV-ECHO version 1.0 [12,13]. Both resources contain de-identified data and were accessed by credentialed investigators under the PhysioNet data-use agreement [14]. The study followed the Strengthening the Reporting of Observational Studies in Epidemiology (STROBE) guidance.

### 2.2. Study Population

**Figure 1:**
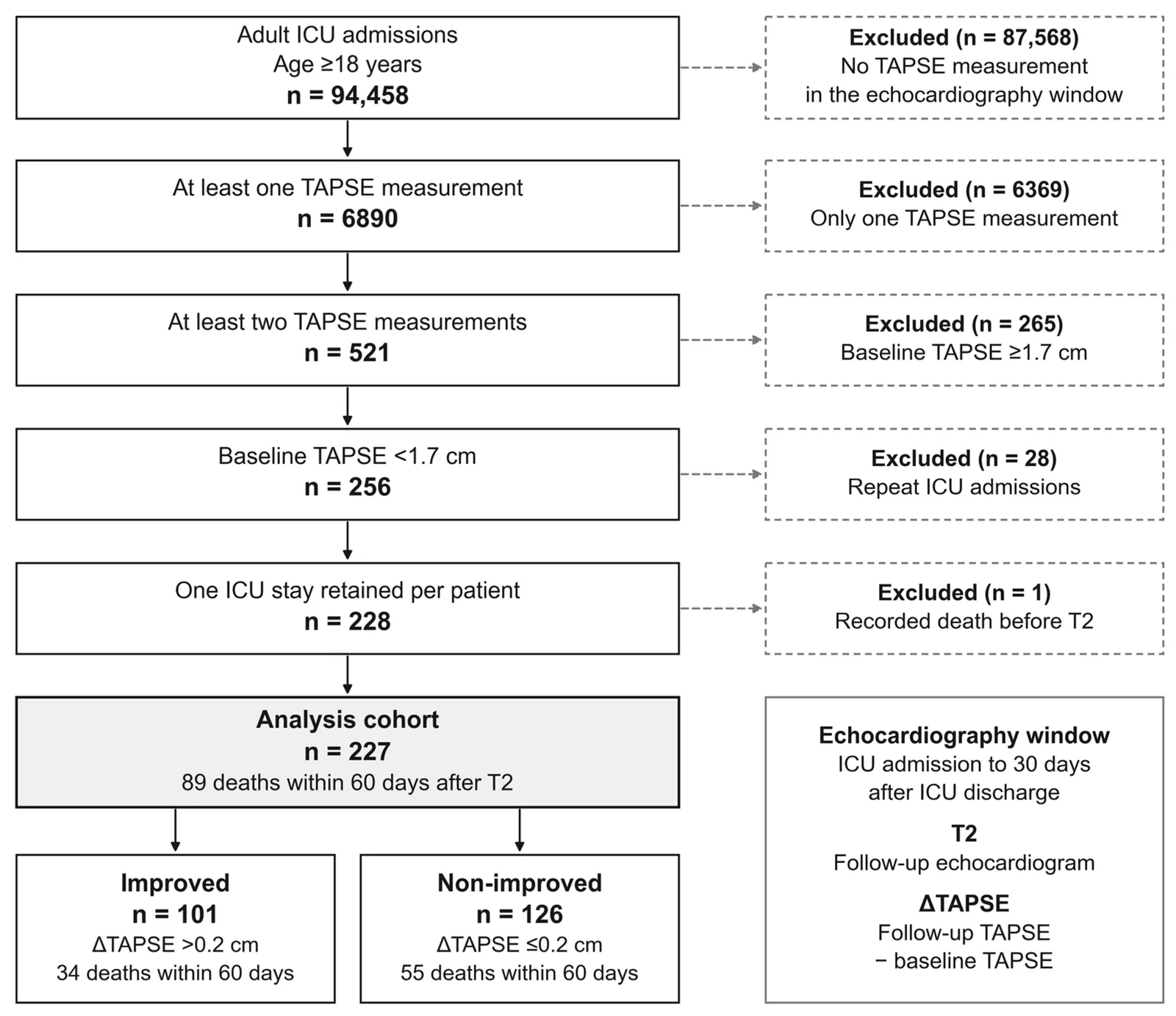
Study flow and serial TAPSE classification. Solid boxes show cohort selection and definitions; dashed boxes show exclusions. Arrows indicate the selection sequence. ICU, intensive care unit; TAPSE, tricuspid annular plane systolic excursion.

We identified adults aged ≥18 years who had at least two TAPSE measurements between ICU admission and 30 days after ICU discharge. The index examination (T1) was the first TAPSE measurement after ICU admission, and the follow-up examination (T2) was the first subsequent TAPSE measurement within this window. Repeat echocardiography was clinically determined. We excluded patients with baseline TAPSE ≥ 1.7 cm and retained one qualifying ICU stay per patient [9,10,11]. One patient with a recorded death time before T2 was excluded, leaving 227 patients (Figure 1). All selected examinations were transthoracic and linked by examination identifier and timestamp.

### 2.3. Serial TAPSE Classification

ΔTAPSE was calculated as follow-up minus baseline TAPSE. Patients were classified as Improved when TAPSE increased by >0.2 cm and Non-improved when the increase was ≤0.2 cm, including stable and decreased values. The >0.2-cm threshold was selected before examining the data as a practical definition of improvement. TAPSE was recorded to one decimal place; therefore, an increase >0.2 cm corresponded to at least 0.3 cm. Alternative thresholds from 0.10 to 0.30 cm, continuous ΔTAPSE, and residualized change were evaluated in sensitivity analyses.

### 2.4. Outcomes

The principal outcome was all-cause death within 60 days after T2, including deaths during and after hospitalization. Death timestamps were obtained from the MIMIC-IV admissions table and death dates from the patients table. Date-only deaths were assigned noon, with start- and end-of-day sensitivity analyses. In the 60-day survival analyses, survivors were censored at day 60. Secondary descriptive outcomes were 28- and 90-day mortality after T2, total index-hospital length of stay, and cumulative time across all ICU stays within that hospitalization.

### 2.5. Covariates and Echocardiographic Measures

Covariates included age, sex, first-day Sequential Organ Failure Assessment (SOFA) score, Charlson comorbidity index, heart failure, pulmonary embolism, acute respiratory distress syndrome, chronic obstructive pulmonary disease, sepsis, creatinine, and lactate. Diagnoses were obtained from records of the index hospitalization. Laboratory covariates were minimum creatinine and maximum lactate in the first ICU day, restricted to samples obtained and reported before T2. Echocardiographic measures included TAPSE, RV and left-ventricular dimensions, left-ventricular ejection fraction, tricuspid-regurgitation jet velocity, pulmonary-artery systolic pressure (PASP), and valvular regurgitation. Measurements were linked within the same examination. PASP was estimated as 4 × (tricuspid-regurgitation jet velocity in m/s)^2^ + 5 mmHg, assuming a right atrial pressure of 5 mmHg. TAPSE/PASP used TAPSE in millimeters and was descriptive, with complete pairs in 147 patients. Mitral and tricuspid regurgitation were dichotomized at moderate severity; grading rules and denominators are in Supplementary Table S1.

We assessed medications and organ support received before T2 during the selected ICU stay. Ventilator pressures were summarized only during invasive ventilation. Coronary procedures described the index hospitalization; coronary artery bypass grafting (CABG) timing used return from the operating room when available, otherwise the procedure date (Supplementary Tables S2 and S3).

Creatinine and lactate were missing in 2 and 34 of 227 patients, respectively. Missing values were handled using multiple imputation (20 datasets). Imputation methods and complete-case results are provided in the Supplementary Tables S4 and S5.

### 2.6. Statistical Analysis

Continuous variables are presented as median [interquartile range] or mean (standard deviation) and were compared using the Mann–Whitney U test or Welch *t* test, respectively. Categorical variables are presented as n/N (%) and were compared using two-sided Fisher exact tests. Paired echocardiographic comparisons used the Wilcoxon signed-rank test. Survival curves were estimated using the Kaplan–Meier method and compared using the log-rank test.

All mortality models used T2 as time zero. The Cox model adjusted for age, sex, Charlson comorbidity index, first-day SOFA score, heart failure, sepsis, chronic obstructive pulmonary disease, and baseline TAPSE. The full model additionally included pulmonary embolism, acute respiratory distress syndrome, creatinine, and lactate. A separate treatment-adjusted model added ICU treatments to the initial model (Supplementary Table S5). Proportional hazards were assessed using Schoenfeld residuals, with a time-varying age effect examined in sensitivity analysis. Adjusted survival curves were averaged over the cohort’s covariate distribution, with pointwise 95% confidence intervals from 1000 bootstrap samples.

Baseline TAPSE was grouped as ≤1.0, >1.0–1.3, and >1.3–<1.7 cm; follow-up TAPSE included an additional ≥1.7-cm group. Cox models used the same clinical covariates described above, with additional adjustment for baseline TAPSE in the follow-up analysis. Overall differences were assessed using log-rank and likelihood-ratio tests; pairwise comparisons used log-rank and Wald tests, respectively, with Holm correction.

Supplementary analyses examined baseline and follow-up TAPSE, continuous ΔTAPSE, and serial change adjusted for baseline or follow-up TAPSE. Additional analyses included restricted cubic splines, alternative ΔTAPSE thresholds, residualized change, logistic regression, interval adjustment, and restriction to the 102 patients with both examinations in the selected ICU stay. A sensitivity analysis excluded patients with T2 < 24 h after ICU admission because first-day SOFA could include measurements after T2.

Analyses used Python 3.12.4 and R 4.5.1; figures were prepared in Python and R. Tests were two-sided, and *p* < 0.05 was considered statistically significant.

During manuscript preparation and revision, the authors used DeepSeek and OpenAI Codex to assist with language editing. The authors determined the manuscript’s argument and interpretation, reviewed the text, and take responsibility for the analyses and final manuscript.

## 3. Results

### 3.1. Cohort Characteristics

The analytic cohort included 227 patients with reduced baseline TAPSE and a clinically obtained follow-up measurement. Mean age was 65.6 years (standard deviation 15.8), and 60.8% were male. The median interval between examinations was 10.0 days [4.6–18.9]. Both examinations occurred in the selected ICU stay for 102 patients; the remaining 125 had T2 after discharge from that stay. Overall, 101 patients (44.5%) were Improved and 126 (55.5%) were Non-improved (Table 1). Further setting details are provided in Supplementary Table S2.

The Improved group had a higher first-day SOFA score (median 7 versus 6; *p* = 0.005) and lower proportions of heart failure (65.3% versus 82.5%; *p* = 0.003) and chronic obstructive pulmonary disease (11.9% versus 24.6%; *p* = 0.017) than the Non-improved group. Age, sex, Charlson comorbidity index, other diagnoses listed in Table 1, and laboratory values did not differ significantly between groups.

Baseline TAPSE was lower in the Improved group (1.10 versus 1.40 cm), whereas follow-up TAPSE was higher (1.80 versus 1.20 cm; both *p* < 0.001). Follow-up TAPSE/PASP and its change were also higher in the Improved group (both *p* < 0.001; Supplementary Figure S1). No significant differences were observed in the examination interval or the other echocardiographic measures, including PASP, LVEF, and MR/TR severity.

### 3.2. Treatments

Most treatment variables did not differ significantly between groups. Compared with the Improved group, patients in the Non-improved group received invasive mechanical ventilation less often (54.8% versus 70.3%; *p* = 0.020) and dobutamine more often (12.7% versus 3.0%; *p* = 0.008). All 19 dobutamine recipients had heart failure diagnoses, 18 had cardiogenic shock diagnoses, and 7 had septic shock diagnoses (Supplementary Table S3).

### 3.3. Clinical Outcomes

**Figure 2:**
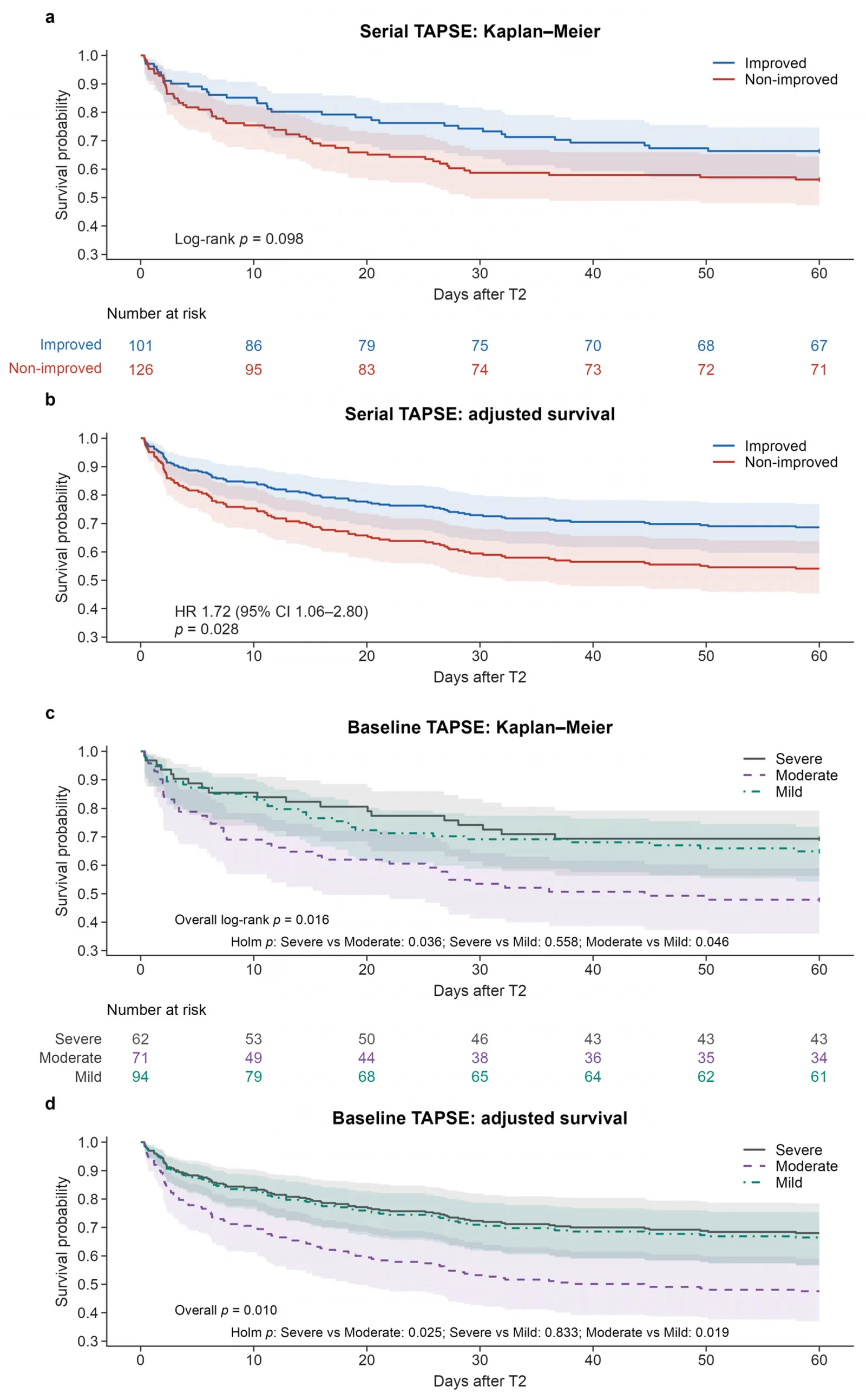
Survival within 60 days after follow-up echocardiography (T2) in 227 patients. (**a**,**b**) Kaplan–Meier and adjusted survival curves by serial TAPSE status: Improved (ΔTAPSE > 0.2 cm) and Non-improved (≤0.2 cm). (**c**,**d**) Corresponding curves by baseline TAPSE: Severe (≤1.0 cm), Moderate (>1.0–1.3 cm), and Mild (>1.3–<1.7 cm). Adjusted curves were averaged over the cohort. Shading shows 95% confidence intervals, with 1000 bootstrap samples used in (**b**,**d**); ticks indicate censoring. Pairwise *p* values are Holm-adjusted. Model covariates and statistical tests are described in Section 2.6. TAPSE, tricuspid annular plane systolic excursion.

There were 89 deaths within 60 days after T2: 34/101 (33.7%) in the Improved group and 55/126 (43.7%) in the Non-improved group. The log-rank *p* value was 0.098 (Figure 2a).

**Figure 3:**
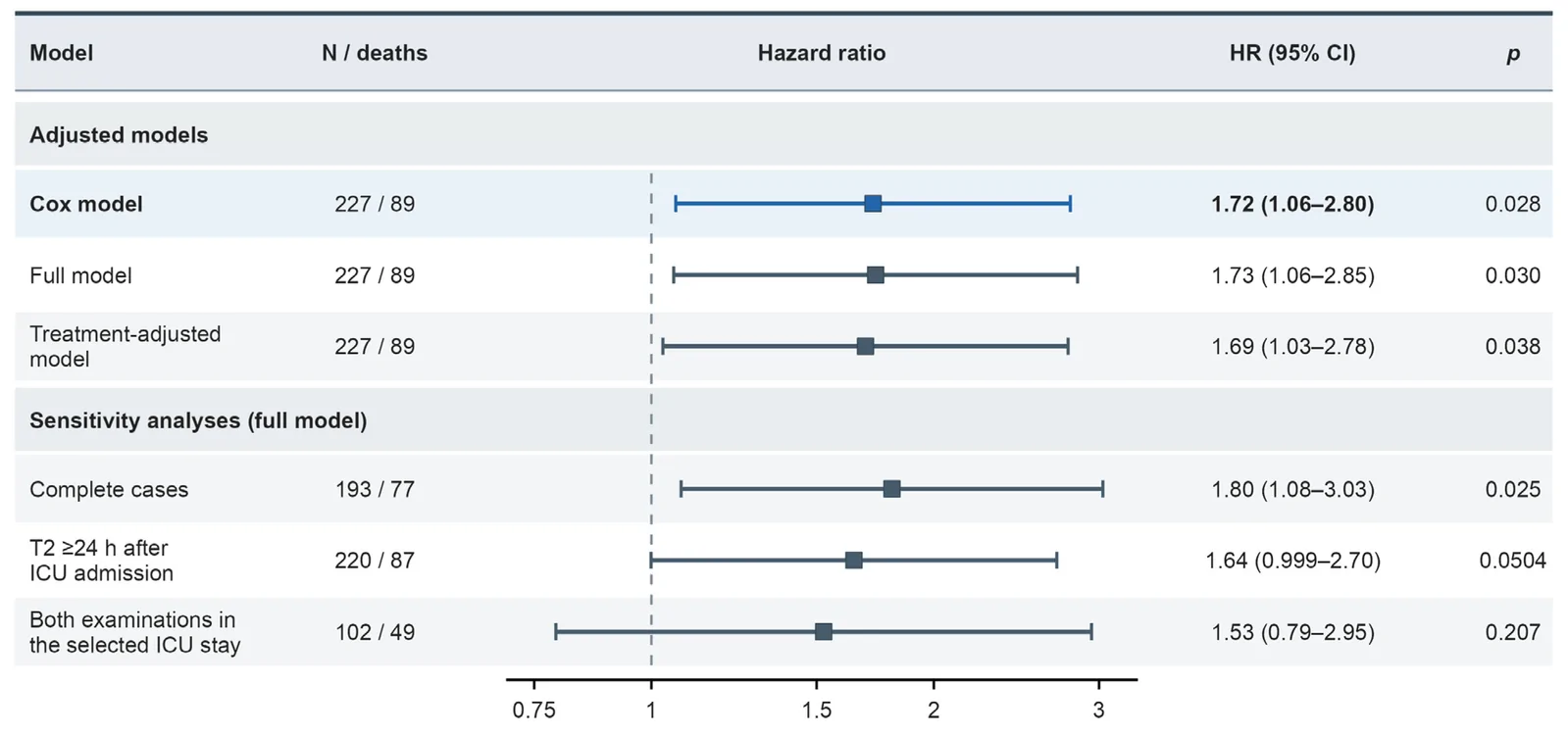
Adjusted associations of Non-improved versus Improved TAPSE status with 60-day mortality after follow-up echocardiography (T2). Squares show hazard ratios (HRs); horizontal bars show 95% confidence intervals (CIs). The dashed line indicates HR = 1. Background shading aids row identification. Model definitions are provided in Supplementary Tables S5 and S4. ICU, intensive care unit; TAPSE, tricuspid annular plane systolic excursion.

In the Cox model, Non-improved status was associated with higher 60-day mortality than Improved status (hazard ratio [HR] 1.72, 95% confidence interval [CI] 1.06–2.80; *p* = 0.028; Figure 2b). Estimates were similar in the full model (HR 1.73, 95% CI 1.06–2.85; *p* = 0.030) and the treatment-adjusted model (HR 1.69, 95% CI 1.03–2.78; *p* = 0.038). Figure 3 summarizes these estimates; full-model coefficients are provided in Supplementary Table S5.

Survival differed across baseline TAPSE strata in both Kaplan–Meier and adjusted analyses (overall *p* = 0.016 and 0.010, respectively; Figure 2c,d). Within the baseline TAPSE > 1.0–1.3 cm stratum, 60-day mortality was 35.5% in Improved patients and 65.0% in Non-improved patients (*p* = 0.017; Supplementary Table S6).

Median total hospital stay was 21.8 days [11.8–37.3], and cumulative ICU stay was 10.4 days [4.7–23.1]. Neither duration differed significantly between the Improved and Non-improved groups. Mortality after T2 was 33.0% at 28 days and 42.7% at 90 days (Table 1). Of the 89 deaths within 60 days, 56 occurred during the index hospitalization, 16 during a later recorded hospitalization, and 17 after discharge without a matched admission; the location of these 17 deaths was unknown (Supplementary Table S2).

When modeled continuously, neither baseline nor follow-up TAPSE was significantly associated with 60-day mortality after adjustment (HR per 0.1 cm, 0.987, *p* = 0.713, and 0.960, *p* = 0.086, respectively). After adjustment for baseline TAPSE, Non-improved status was associated with higher mortality (HR 1.72, 95% CI 1.06–2.80; *p* = 0.028), whereas continuous ΔTAPSE was not statistically significant (HR 0.960 per 0.1 cm; *p* = 0.093). After adjustment for follow-up TAPSE, neither Non-improved status (HR 1.36, 95% CI 0.78–2.36; *p* = 0.275) nor continuous ΔTAPSE (*p* = 0.982) was statistically significant (Supplementary Table S7). Restricted cubic spline analysis showed no significant overall or nonlinear association between ΔTAPSE and 60-day mortality (overall *p* = 0.157; nonlinearity *p* = 0.388; Supplementary Figure S2). Survival did not differ significantly across follow-up TAPSE groups before or after adjustment (overall *p* = 0.414 and 0.204, respectively; Supplementary Figure S3).

### 3.4. Sensitivity Analyses

Across alternative ΔTAPSE thresholds, full-model HRs ranged from 1.65 to 1.73 (Supplementary Table S8). Estimates were similar with alternative death-date assignments, complete cases, and a time-varying age effect. Excluding seven patients with T2 within 24 h of ICU admission yielded a full-model HR of 1.64 (95% CI 0.999–2.705; *p* = 0.0504; Supplementary Table S4).

In full-model sensitivity analyses, residualized change was not statistically significant (HR 1.21 per standard-deviation worse-than-expected change, 95% CI 0.96–1.53; *p* = 0.111). The ICU-only estimate was HR 1.53 (95% CI 0.79–2.95; *p* = 0.207), and the landmark logistic estimate was OR 1.84 (95% CI 0.95–3.57; *p* = 0.069; Supplementary Table S4).

## 4. Discussion

In this cohort of critically ill adults with reduced baseline TAPSE and clinically indicated repeat echocardiography, non-improved serial TAPSE status was associated with higher 60-day mortality after the follow-up examination. These findings suggest that serial TAPSE may help assess prognosis in this population.

Bedside echocardiography provides several measures of RV systolic function, including TAPSE, RV fractional area change, tissue Doppler S′, and RV strain [9,10,11]. TAPSE is simple to obtain using M-mode imaging. Although TAPSE/PASP has physiological and prognostic value, PASP estimation requires a measurable tricuspid regurgitation signal [15,16]. Paired PASP measurements were available in 147 patients (64.8%) in our cohort, making TAPSE more consistently available for serial assessment. We therefore used the change in TAPSE between examinations to classify patients as Improved or Non-improved. The >0.2-cm threshold was selected before examining the data as a practical definition of improvement. Measurement variability may affect this classification, particularly in critically ill patients [17,18,19].

Longitudinal RV assessment has been studied in chronic heart failure and pulmonary arterial hypertension, with repeat examinations months after baseline [20,21]. In septic shock, a study focused on TAPSE at reassessment linked RV dysfunction at seven days to in-hospital mortality [22]. Our study extends this work to within-patient TAPSE changes over a median of 10 days in critically ill patients with varied underlying diseases, suggesting that short-term TAPSE changes may also have clinical relevance.

Short-term TAPSE changes may reflect changes in ventricular contraction and loading conditions [11,17]. Fluid therapy, vasoactive drugs, and mechanical ventilation can alter these conditions between examinations [23,24]. Underlying disease may also influence the course of RV function. RV dysfunction has prognostic value in heart failure [25,26,27] and pulmonary hypertension associated with chronic lung disease [28,29]. In our cohort, heart failure and COPD were more common in the Non-improved group. Greater dobutamine use in this group may reflect this disease background, as all recipients had heart-failure diagnoses and most had cardiogenic-shock diagnoses.

In our cohort, the predefined change pattern remained associated with mortality after adjustment for baseline TAPSE, suggesting that repeat assessment may add information beyond the initial examination. The clinical meaning of an absolute TAPSE value may vary with underlying disease and loading conditions [11], which may help explain the lack of a significant association for follow-up TAPSE alone. Studies linking follow-up RV dysfunction in septic shock and recovery of TAPSE in chronic heart failure to prognosis also support the relevance of reassessment [20,22]. Continuous ΔTAPSE was not significantly associated with mortality, and adjustment for follow-up TAPSE weakened the binary association. Larger prospective studies should assess how the magnitude of TAPSE change relates to prognosis and whether serial change adds prognostic information beyond the follow-up measurement.

Rako et al. described RV adaptation in terms of matching contractility to pulmonary vascular load [30]. The TAPSE/PASP ratio has been validated against invasive measures of RV–pulmonary arterial coupling in pulmonary hypertension and heart failure [31,32], and its prognostic value has been reported in these conditions and septic shock [33,34,35]. In our 147 patients with paired measurements, median estimated PASP changed from 38.6 to 41.0 mmHg in the Improved group (*p* = 0.325) and from 39.8 to 38.6 mmHg in the Non-improved group (*p* = 0.717). Over the same interval, median TAPSE/PASP increased from 0.31 to 0.43 mm/mmHg in the Improved group and decreased from 0.33 to 0.28 mm/mmHg in the Non-improved group. TAPSE was used both to define the groups and as the ratio’s numerator. Further studies are needed to determine whether these ratio changes reflect changes in RV–pulmonary arterial coupling and relate to prognosis.

This study has several limitations. First, the retrospective single-center design and inclusion of only patients who survived to repeat echocardiography may limit generalizability [36]. Examination timing was not standardized, specific indications were unavailable, and 125 follow-up examinations occurred after discharge from the selected ICU stay. Second, TAPSE reflects longitudinal annular motion and is affected by loading conditions, imaging angle, cursor placement, and image quality [11,17]. Serial measurements of other RV indices were unavailable, and paired pressure and fluid data were limited. PASP estimation also assumed a fixed right atrial pressure. Third, lower baseline TAPSE in the Improved group may have contributed to regression to the mean despite adjustment [37]. Hospital diagnosis codes did not establish whether each condition was present before T2, leaving possible residual confounding. Finally, the sample size limited the precision of estimates, particularly in the ICU-only analyses.

## 5. Conclusions

In this exploratory study of critically ill adults with reduced baseline TAPSE, non-improved serial TAPSE status was associated with higher 60-day mortality after repeat echocardiography. However, serial change did not show additional prognostic information beyond the follow-up TAPSE value.

## Figures and Tables

**Table 1:** Patient characteristics, echocardiographic measures, treatments, and outcomes by serial TAPSE status.

Variable	Overall (n = 227)	Improved (n = 101)	Non-Improved (n = 126)	*p*-Value
**Demographics**				
Age, mean (SD), years	65.6 (15.8)	65.2 (15.2)	65.9 (16.3)	0.738
Male sex, n (%)	138/227 (60.8%)	66/101 (65.3%)	72/126 (57.1%)	0.221
Charlson Comorbidity Index, median [IQR]	6.0 [4.0–8.0]	5.0 [4.0–8.0]	6.0 [4.0–8.0]	0.468
**Disease Severity**				
SOFA score, median [IQR]	6.0 [4.0–9.0]	7.0 [5.0–11.0]	6.0 [3.0–8.0]	0.005
**Echocardiographic Parameters**				
**Right-Heart Echocardiographic Measures**				
Index TAPSE, median [IQR], cm	1.30 [1.00–1.50]	1.10 [0.90–1.40]	1.40 [1.10–1.50]	<0.001
Follow-up TAPSE, median [IQR], cm	1.40 [1.10–1.70]	1.80 [1.40–2.10]	1.20 [1.00–1.30]	<0.001
ΔTAPSE, median [IQR], cm	0.20 [−0.10–0.55]	0.60 [0.40–0.90]	−0.10 [−0.30–0.10]	<0.001
Echo interval, median [IQR], days	10.02 [4.58–18.88]	11.03 [5.11–19.95]	8.11 [4.02–18.52]	0.095
RV diastolic dimension, median [IQR], cm	4.30 [3.62–4.90] (n = 210)	4.30 [3.70–4.90] (n = 97)	4.20 [3.60–4.90] (n = 113)	0.672
RV/LV diameter ratio, median [IQR]	0.86 [0.73–1.08] (n = 197)	0.90 [0.74–1.12] (n = 89)	0.83 [0.73–1.01] (n = 108)	0.193
PASP, median [IQR], mmHg	38.6 [30.0–48.6] (n = 192)	35.3 [28.0–46.0] (n = 82)	38.6 [32.0–48.6] (n = 110)	0.292
TR jet velocity, median [IQR], m/s	2.90 [2.50–3.30] (n = 192)	2.75 [2.40–3.20] (n = 82)	2.90 [2.60–3.30] (n = 110)	0.292
TAPSE/PASP ratio, median [IQR], mm/mmHg	0.326 [0.228–0.439] (n = 192)	0.319 [0.187–0.432] (n = 82)	0.336 [0.247–0.439] (n = 110)	0.106
Follow-up PASP, median [IQR], mmHg	38.6 [32.0–48.6] (n = 170)	41.0 [30.0–49.9] (n = 75)	38.6 [32.0–48.6] (n = 95)	0.942
Follow-up TR jet velocity, median [IQR], m/s	2.90 [2.60–3.30] (n = 170)	3.00 [2.50–3.35] (n = 75)	2.90 [2.60–3.30] (n = 95)	0.942
Follow-up TAPSE/PASP ratio, median [IQR], mm/mmHg	0.351 [0.238–0.463] (n = 170)	0.435 [0.317–0.638] (n = 75)	0.285 [0.216–0.392] (n = 95)	<0.001
ΔTAPSE/PASP ratio, median [IQR], mm/mmHg	0.027 [−0.051–0.151] (n = 147)	0.131 [0.032–0.228] (n = 63)	−0.030 [−0.097–0.040] (n = 84)	<0.001
**LV Function**				
LVEF, median [IQR], %	40.0 [25.0–55.0] (n = 202)	40.0 [28.2–55.0] (n = 90)	37.0 [25.0–55.0] (n = 112)	0.243
LV diastolic dimension, median [IQR], cm	4.80 [4.20–5.35] (n = 203)	4.60 [4.25–5.20] (n = 91)	4.85 [4.20–5.50] (n = 112)	0.156
Fractional shortening, median [IQR], %	23.0 [15.0–32.8] (n = 154)	24.0 [14.0–34.0] (n = 70)	21.5 [15.8–32.0] (n = 84)	0.882
**Cardiac Chambers**				
LA diameter, median [IQR], cm	3.90 [3.40–4.40] (n = 188)	3.90 [3.25–4.40] (n = 84)	3.95 [3.50–4.50] (n = 104)	0.344
**Valvular Function**				
Moderate/severe MR, n (%)	52/204 (25.5%)	19/93 (20.4%)	33/111 (29.7%)	0.148
Moderate/severe TR, n (%)	91/210 (43.3%)	38/94 (40.4%)	53/116 (45.7%)	0.485
**Etiology and Comorbidities**				
Heart failure, n (%)	170/227 (74.9%)	66/101 (65.3%)	104/126 (82.5%)	0.003
Sepsis, n (%)	90/227 (39.6%)	39/101 (38.6%)	51/126 (40.5%)	0.787
Pneumonia, n (%)	73/227 (32.2%)	28/101 (27.7%)	45/126 (35.7%)	0.253
COPD, n (%)	43/227 (18.9%)	12/101 (11.9%)	31/126 (24.6%)	0.017
Pulmonary embolism, n (%)	23/227 (10.1%)	9/101 (8.9%)	14/126 (11.1%)	0.662
ARDS, n (%)	21/227 (9.3%)	12/101 (11.9%)	9/126 (7.1%)	0.254
**Admission Laboratory Values (first 24 h)**				
Creatinine (min), median [IQR], mg/dL	1.30 [0.90–1.90] (n = 225)	1.20 [0.90–1.90] (n = 100)	1.30 [0.90–1.90] (n = 125)	0.428
Lactate (max), median [IQR], mmol/L	2.90 [1.70–5.10] (n = 193)	2.90 [1.60–5.10] (n = 89)	2.80 [1.70–4.88] (n = 104)	0.940
Bicarbonate (min), mean (SD), mEq/L	20.3 (5.5) (n = 225)	20.2 (5.3) (n = 100)	20.4 (5.6) (n = 125)	0.792
WBC (max), median [IQR], ×10^3^/μL	14.2 [9.9–18.9] (n = 225)	13.9 [9.5–18.8] (n = 100)	14.4 [10.3–19.6] (n = 125)	0.506
Hemoglobin (min), mean (SD), g/dL	9.93 (2.51) (n = 225)	9.90 (2.55) (n = 100)	9.96 (2.50) (n = 125)	0.851
Troponin T (max), median [IQR], ng/mL	0.29 [0.07–1.02] (n = 99)	0.20 [0.07–0.90] (n = 45)	0.36 [0.07–1.06] (n = 54)	0.733
**ICU Treatments before T2**				
Invasive mechanical ventilation, n (%)	140/227 (61.7%)	71/101 (70.3%)	69/126 (54.8%)	0.020
IMV duration among ventilated patients, median [IQR], days	2.80 [1.27–7.43] (n = 140)	2.75 [1.35–9.17] (n = 71)	2.96 [1.14–6.21] (n = 69)	0.391
PEEP (max), median [IQR], cmH_2_O	10.0 [6.2–12.0] (n = 140)	10.0 [7.8–13.0] (n = 71)	10.0 [5.5–12.0] (n = 69)	0.085
Plateau pressure (max), median [IQR], cmH_2_O	23.0 [19.1–27.0] (n = 135)	23.0 [19.9–29.0] (n = 68)	22.0 [18.3–26.5] (n = 67)	0.149
Arterial P/F ratio (min), median [IQR]	170.0 [98.1–287.0] (n = 123)	145.7 [88.0–282.8] (n = 63)	199.8 [114.2–287.6] (n = 60)	0.101
Norepinephrine, n (%)	80/227 (35.2%)	39/101 (38.6%)	41/126 (32.5%)	0.402
Vasopressin, n (%)	46/227 (20.3%)	20/101 (19.8%)	26/126 (20.6%)	1.000
Dopamine, n (%)	4/227 (1.8%)	3/101 (3.0%)	1/126 (0.8%)	0.326
Epinephrine, n (%)	35/227 (15.4%)	14/101 (13.9%)	21/126 (16.7%)	0.585
Phenylephrine, n (%)	56/227 (24.7%)	25/101 (24.8%)	31/126 (24.6%)	1.000
Any vasopressor, n (%)	94/227 (41.4%)	42/101 (41.6%)	52/126 (41.3%)	1.000
Dobutamine, n (%)	19/227 (8.4%)	3/101 (3.0%)	16/126 (12.7%)	0.008
IV furosemide, n (%)	91/227 (40.1%)	37/101 (36.6%)	54/126 (42.9%)	0.414
Active CRRT, n (%)	52/227 (22.9%)	23/101 (22.8%)	29/126 (23.0%)	1.000
**Procedures during Index Hospitalization**				
Percutaneous coronary intervention (PCI), n (%)	18/227 (7.9%)	7/101 (6.9%)	11/126 (8.7%)	0.806
CABG, n (%)	27/227 (11.9%)	10/101 (9.9%)	17/126 (13.5%)	0.537
**Clinical Outcomes**				
Cumulative ICU length of stay, median [IQR], days	10.38 [4.66–23.05]	9.11 [4.27–22.06]	11.20 [5.00–23.06]	0.772
Hospital length of stay, median [IQR], days	21.80 [11.79–37.31]	20.84 [11.07–37.96]	24.26 [12.31–36.66]	0.645
28-day mortality, n (%)	75/227 (33.0%)	25/101 (24.8%)	50/126 (39.7%)	0.023
60-day mortality, n (%)	89/227 (39.2%)	34/101 (33.7%)	55/126 (43.7%)	0.135
90-day mortality, n (%)	97/227 (42.7%)	38/101 (37.6%)	59/126 (46.8%)	0.179

Values are median [interquartile range], mean (standard deviation), or n/N (%). Available counts are shown where values are missing. Comparisons used Mann–Whitney U, Welch t, or Fisher exact tests, respectively. Missing values in Table 1 were not imputed; *p* values are unadjusted for multiple comparisons. PASP was estimated from TR velocity, assuming a right atrial pressure of 5 mmHg. TAPSE/PASP used measurements from the same examination. MR and TR percentages were based on available grades. We used laboratory results from the first ICU day that were available before T2. We assessed medications and organ support received before T2 during the selected ICU stay. Ventilation duration and pressures were summarized for the 140 patients who received invasive ventilation. Plateau pressure was missing in 5 patients. PCI and CABG were recorded during the index hospitalization; CABG timing is shown in Supplementary Table S2. Hospital stay was measured from admission to discharge. Total ICU time included all ICU stays during the same hospitalization. Abbreviations: ARDS, acute respiratory distress syndrome; CABG, coronary artery bypass grafting; COPD, chronic obstructive pulmonary disease; CRRT, continuous renal replacement therapy; ICU, intensive care unit; IMV, invasive mechanical ventilation; IV, intravenous; LA, left atrium; LV, left ventricle; LVEF, left-ventricular ejection fraction; MR, mitral regurgitation; PASP, pulmonary-artery systolic pressure; PEEP, positive end-expiratory pressure; PCI, percutaneous coronary intervention; P/F, arterial PaO_2_/FiO_2_; RV, right ventricle; SOFA, Sequential Organ Failure Assessment; T1/T2, baseline/follow-up echocardiography; TAPSE, tricuspid annular plane systolic excursion; TR, tricuspid regurgitation; WBC, white blood cell count. Gray shading and bold text identify table headers and section headings.

## Data Availability

MIMIC-IV and MIMIC-IV-ECHO are available to credentialed users through PhysioNet (https://physionet.org). The analysis code is available from the corresponding author upon reasonable request.

## Supplementary Materials

The following supporting information can be downloaded at: https://www.mdpi.com/article/10.3390/jcm15197753/s1, Table S1: Available data and paired measurements; Table S2: Examination setting, death setting, and CABG timing; Table S3: Treatment windows and paired pressure and fluid context; Table S4: Timing and model sensitivity analyses; Table S5: Model definitions and full Cox and logistic regression results; Table S6: Baseline TAPSE strata and 60-day mortality after T2; Table S7: TAPSE measures and 60-day mortality; Table S8: Alternative fixed ΔTAPSE improvement thresholds; Figure S1: Serial TAPSE, estimated PASP, and TAPSE/PASP; Figure S2: ΔTAPSE distribution and continuous association; Figure S3: Follow-up TAPSE and 60-day survival.
