# Supplementary material for: Serial TAPSE and Subsequent 60-Day Mortality Among Critically Ill Adults with Reduced Baseline TAPSE: A MIMIC-IV-ECHO Cohort Study
Source: J Clin Med. 2026 Oct 8;15(19):7753. doi: 10.3390/jcm15197753 (PMC13665873; doi:10.3390/jcm15197753)
Supplement: Supplementary file 1 [file jcm-15-07753-s001.zip › Supplement_FINAL.pdf]

# Supplementary Materials

## Serial TAPSE and Subsequent 60-Day Mortality among Critically Ill Adults with Reduced Baseline TAPSE: A MIMIC-IV-ECHO Cohort Study

### Table S1. Available data and paired measurements

**Panel A. Available data**

| Measure | Overall available/227 | Improved available/101 | Non-improved available/126 |
|---|---|---|---|
| RV diastolic dimension, cm | 210/227 | 97/101 | 113/126 |
| RV/LV diastolic dimension ratio | 197/227 | 89/101 | 108/126 |
| Estimated baseline PASP, mmHg | 192/227 | 82/101 | 110/126 |
| Baseline TR velocity, m/s | 192/227 | 82/101 | 110/126 |
| Baseline TAPSE/estimated PASP, mm/mmHg | 192/227 | 82/101 | 110/126 |
| Reported baseline LVEF, % | 202/227 | 90/101 | 112/126 |
| LV diastolic dimension, cm | 203/227 | 91/101 | 112/126 |
| Fractional shortening, % | 154/227 | 70/101 | 84/126 |
| LA diameter, cm | 188/227 | 84/101 | 104/126 |
| Estimated follow-up PASP, mmHg | 170/227 | 75/101 | 95/126 |
| Follow-up TR velocity, m/s | 170/227 | 75/101 | 95/126 |
| Follow-up TAPSE/estimated PASP, mm/mmHg | 170/227 | 75/101 | 95/126 |
| ΔTAPSE/estimated PASP, mm/mmHg | 147/227 | 63/101 | 84/126 |
| Moderate or greater MR | 204/227 | 93/101 | 111/126 |
| Moderate or greater TR | 210/227 | 94/101 | 116/126 |
| Creatinine minimum, mg/dL | 225/227 | 100/101 | 125/126 |
| Lactate maximum, mmol/L | 193/227 | 89/101 | 104/126 |
| Bicarbonate minimum, mEq/L | 225/227 | 100/101 | 125/126 |
| WBC maximum, $\times 10^3/\mu L$ | 225/227 | 100/101 | 125/126 |
| Hemoglobin minimum, g/dL | 225/227 | 100/101 | 125/126 |
| Troponin T maximum, ng/mL | 99/227 | 45/101 | 54/126 |
| Arterial P/F ratio minimum, mmHg | 123/227 | 63/101 | 60/126 |
| IMV duration among ventilated patients, days | 140/227 | 71/101 | 69/126 |
| Maximum PEEP during IMV, $cmH_2O$ | 140/227 | 71/101 | 69/126 |
| Maximum plateau pressure during IMV, $cmH_2O$ | 135/227 | 68/101 | 67/126 |

**Panel B. Paired PASP and TAPSE/PASP**

| Measure and time | Improved (n = 63) | Non-improved (n = 84) | *p* |
|---|---|---|---|
| PASP T1 | 38.64 [32.04–54.00] | 39.82 [33.63–49.23] | 0.661 |
| PASP T2 | 41.00 [30.00–51.24] | 38.64 [34.16–48.56] | 0.975 |
| PASP change | 2.12 [-6.06–9.12] | -0.98 [-9.44–8.08] | 0.359 |
| TAPSE/PASP T1 | 0.309 [0.171–0.385] | 0.326 [0.234–0.425] | 0.140 |
| TAPSE/PASP T2 | 0.435 [0.292–0.543] | 0.283 [0.218–0.389] | <0.001 |
| TAPSE/PASP change | 0.131 [0.032–0.228] | -0.030 [-0.097–0.040] | <0.001 |

T1 and T2 denote baseline and follow-up echocardiography. Panel A lists patients with available data. Echocardiographic values came from the same examination, matched by examination ID and time. MR and TR were grouped as at least moderate or less than moderate; mild-to-moderate grades were included in the latter group. Ungradable values were treated as missing. All 202 LVEF values came from the reported value or, if unavailable, the biplane value.

Ventilation measures applied to 140 patients; plateau pressure was missing in 5. Other Table 1 variables had complete data. Descriptive values were not imputed.

Panel B shows medians [IQR] for 147 patients with paired measurements. PASP = 4 × TR velocity$^2$ + 5 mmHg, with TR velocity in m/s. TAPSE/PASP used TAPSE in mm. Missing TR velocity meant PASP and TAPSE/PASP were unavailable. Calculations used unrounded values.

Between-group comparisons used the Mann–Whitney U test. Within-group Wilcoxon *p* values were 0.325 and 0.717 for PASP, and <0.001 and 0.016 for TAPSE/PASP (Improved and Non-improved, respectively). Wilcoxon tests used an asymptotic approximation with continuity correction; differences were rounded to 3 decimals for PASP and 6 for TAPSE/PASP to handle ties.

## Table S2. Examination setting, death setting, and CABG timing

### Panel A. Examination setting

| Classification | n/N (%) |
|---|---|
| T1 and T2 matched to the same hospitalization | 186/227 (81.9%) |
| T1 and T2 matched to different hospitalizations | 25/227 (11.0%) |
| At least one examination unmatched to an admission interval | 16/227 (7.0%) |
| T2 within the index hospitalization | 183/227 (80.6%) |
| T2 during a later recorded hospitalization | 30/227 (13.2%) |
| T2 outside any matched hospitalization interval | 14/227 (6.2%) |
| T2 labeled Inpatient in the echo source | 215/227 (94.7%) |
| T2 labeled Outpatient in the echo source | 12/227 (5.3%) |
| T2 during the selected index ICU stay | 102/227 (44.9%) |
| T2 during another recorded ICU stay | 42/227 (18.5%) |
| T2 outside any matched ICU interval | 83/227 (36.6%) |

### Panel B. Death setting

| 60-day death setting | Overall deaths (n = 89) | Improved deaths (n = 34) | Non-improved deaths (n = 55) |
|---|---|---|---|
| Index hospitalization | 56 (62.9%) | 23 (67.6%) | 33 (60.0%) |
| Later recorded hospitalization | 16 (18.0%) | 5 (14.7%) | 11 (20.0%) |
| After discharge, no matched admission | 17 (19.1%) | 6 (17.6%) | 11 (20.0%) |

### Panel C. CABG timing

| CABG timing (n = 27) | Patients, n |
|---|---|
| Return from operating room recorded | 18 |
| Procedure date only | 9 |
| Both examinations after surgery | 20 |
| T1 before and T2 after surgery | 6 |
| Both examinations before surgery | 1 |
| T2 after surgery | 26 |

Hospital and ICU settings were assigned using admission and discharge times; echo source labels are shown separately. Of 12 outpatient-labeled examinations, 11 had no matched hospitalization and one overlapped the index hospitalization. Three inpatient-labeled examinations had no match. Reasons for repeat echocardiography were unavailable in the structured records.

Death percentages use deaths as denominators. The location of the 17 deaths after discharge without a matched admission was unknown.

CABG timing used return from the operating room when available, otherwise the procedure date. Of 18 recorded returns, 17 were on the procedure date and one was after midnight. PCI was an index-hospitalization indicator (18 patients), without timing relative to T2.

## Table S3. Treatment windows and paired pressure and fluid context

### Panel A. Dobutamine use

| **Dobutamine context among 19 recipients** | **n** |
|---|---|
| Improved / Non-improved | 3 / 16 |
| Heart-failure diagnosis | 19 |
| Cardiogenic-shock diagnosis, including postprocedural | 18 |
| Septic-shock diagnosis, including postprocedural | 7 |
| Both shock diagnosis categories | 6 |
| Concurrent vasopressor exposure | 13 |
| Dobutamine started before T1 / between T1 and T2 | 13 / 6 |
| CABG during index hospitalization | 3 |

### Panel B. Pressure and fluid changes

| **Paired change correlated with ΔTAPSE** | **Pairs** | **Spearman ρ** | **Raw *p*** | **Holm *p*** |
|---|---|---|---|---|
| ΔPEEP | 32 | -0.108 | 0.557 | 0.887 |
| Δplateau pressure | 17 | 0.199 | 0.444 | 0.887 |
| Δrecorded 24-h input | 28 | -0.147 | 0.457 | 0.457 |
| Δrecorded 24-h urine | 30 | 0.232 | 0.218 | 0.436 |
| Δrecorded 24-h input minus output | 14 | -0.596 | 0.024 | 0.073 |

Treatments were recorded before T2 during the selected ICU stay; overlapping drug administrations were included. Any vasopressor included norepinephrine, vasopressin, dopamine, epinephrine, or phenylephrine. Dobutamine was assessed separately. IMV duration used merged ventilation intervals and excluded tracheostomy-only intervals. CRRT required active treatment records. Diagnoses were index-hospitalization codes; onset times, drug indications, and treatment protocols were unavailable.

Pressures were the nearest preceding values within six hours of each examination during invasive ventilation in the same ICU stay. Unavailable ICU measurements were treated as missing. Fluid measurements covered the preceding 24 hours and required full ICU coverage. Input volume was allocated by infusion overlap, assuming a constant rate within each infusion segment; output used recorded event times. Irrigation, residual-volume measurements, insufficiently timed records, and eight negative outputs (seven chest-drain and one Jackson–Pratt entry) were excluded. Recorded input minus output depends on documentation completeness.

Changes were T2 minus T1. Correlations used Spearman coefficients, with Holm adjustment for the two pressure and three fluid comparisons separately.

**Table S4. Timing and model sensitivity analyses**

| Analysis | n / deaths | Type | Estimate (95% CI) | *p* |
|---|---|---|---|---|
| Date-only deaths at start of day; C + T1 | 227 / 89 | HR | 1.722 (1.061–2.794) | 0.028 |
| Date-only deaths at end of day; C + T1 | 227 / 89 | HR | 1.722 (1.061–2.795) | 0.028 |
| Add log examination interval; C + T1 | 227 / 89 | HR | 1.709 (1.049–2.782) | 0.031 |
| Add age × log(1 + time); C + T1 | 227 / 89 | HR | 1.821 (1.119–2.965) | 0.016 |
| Full model, complete cases | 193 / 77 | HR | 1.805 (1.076–3.028) | 0.025 |
| Full model, exclude T2 <24 h after ICU entry | 220 / 87 | HR | 1.644 (0.999–2.705) | 0.0504 |
| Full model, age × log(1 + time) | 227 / 89 | HR | 1.822 (1.107–2.999) | 0.018 |
| Residualized change, per SD; C | 227 / 89 | HR | 1.210 (0.962–1.522) | 0.104 |
| Residualized change, per SD; F | 227 / 89 | HR | 1.211 (0.957–1.532) | 0.111 |
| Both examinations in index ICU; C + T1 | 102 / 49 | HR | 1.518 (0.803–2.869) | 0.199 |
| Both examinations in index ICU; F + T1 | 102 / 49 | HR | 1.526 (0.791–2.945) | 0.207 |
| Index-ICU-only, full complete cases | 93 / 45 | HR | 1.582 (0.799–3.134) | 0.189 |
| 60-day T2-origin logistic; F + T1 | 227 / 89 | OR | 1.844 (0.953–3.568) | 0.069 |
| Residualized change logistic, per SD; F | 227 / 89 | OR | 1.253 (0.918–1.711) | 0.155 |
| Index-ICU-only logistic; F + T1 | 102 / 49 | OR | 1.557 (0.614–3.947) | 0.351 |

C includes age (per 10 years), male sex, Charlson comorbidity index, first-day SOFA score, heart failure, sepsis, and COPD. F adds pulmonary embolism, ARDS, minimum creatinine, and maximum lactate. TAPSE and ΔTAPSE are expressed per 0.1 cm. Diagnoses refer to the index hospitalization; onset dates were unavailable.

Except for residualized-change rows, estimates compare Non-improved with Improved patients. ICU-only analyses required both examinations during the selected ICU stay. Date-only deaths were assigned noon; sensitivity analyses used 00:00 or 23:59:59. Interval adjustment used log(days between T1 and T2). The age sensitivity model added age × log(1 + days after T2).

Residualized change was the negative standardized residual from ΔTAPSE regressed on baseline TAPSE and examination interval, with an intercept. Higher values indicate less improvement than expected. ICU-only models used the relevant patients from the 20 full-cohort imputed datasets. The early-T2 exclusion analysis used a separate imputation run (Table S5). HR, hazard ratio; OR, odds ratio; CI, confidence interval.

**Table S5. Model definitions and full Cox and logistic regression results**

| Variable | Cox HR (95% CI) | *p* | Logistic OR (95% CI) | *p* |
|---|---|---|---|---|
| Age, per 10 years | 1.122 (0.931–1.353) | 0.225 | 1.128 (0.880–1.447) | 0.341 |
| Male sex | 0.720 (0.468–1.108) | 0.135 | 0.721 (0.395–1.316) | 0.286 |
| Charlson index, per point | 1.059 (0.969–1.156) | 0.206 | 1.111 (0.972–1.270) | 0.122 |
| First-day SOFA, per point | 1.058 (0.986–1.137) | 0.119 | 1.066 (0.971–1.171) | 0.177 |
| Heart failure | 0.705 (0.404–1.230) | 0.218 | 0.629 (0.293–1.351) | 0.234 |
| Sepsis | 1.804 (1.137–2.861) | 0.012 | 2.113 (1.131–3.949) | 0.019 |
| COPD | 1.909 (1.103–3.302) | 0.021 | 2.166 (0.973–4.821) | 0.058 |
| Pulmonary embolism | 1.241 (0.617–2.498) | 0.545 | 0.900 (0.324–2.499) | 0.839 |
| ARDS | 1.558 (0.763–3.181) | 0.223 | 2.202 (0.778–6.238) | 0.137 |
| Creatinine, per mg/dL | 0.937 (0.770–1.141) | 0.519 | 0.945 (0.738–1.211) | 0.656 |
| Lactate, per mmol/L | 1.012 (0.940–1.091) | 0.749 | 1.015 (0.909–1.133) | 0.794 |
| Baseline TAPSE, per 0.1 cm | 0.955 (0.881–1.036) | 0.269 | 0.952 (0.850–1.066) | 0.394 |
| Non-improved versus Improved | 1.734 (1.056–2.846) | 0.030 | 1.844 (0.953–3.568) | 0.069 |

All analyses used 227 patients and 89 deaths within 60 days after T2. All listed variables entered each full model together. C and F are defined in Table S4. The Cox model used C + baseline TAPSE + serial status (9 coefficients); the full model used F instead of C (13). The treatment model added IMV, any vasopressor, dobutamine, intravenous furosemide, and active CRRT to the Cox model (14).

Cox models used the Efron method for ties. Missing creatinine and lactate were imputed after log transformation using predictive mean matching: 20 datasets, 20 burn-in cycles, five cycles between datasets, and five donors. Predictors included clinical covariates, baseline and follow-up TAPSE, serial status, the death indicator, and Nelson–Aalen cumulative hazard. Cox and logistic estimates used the same imputed datasets and were pooled using Rubin’s rules, with normal-approximation confidence intervals and *p* values. Laboratory values entered the models in their original units. HR, hazard ratio; OR, odds ratio; CI, confidence interval.

**Table S6. Baseline TAPSE strata and 60-day mortality after T2**

| Baseline TAPSE | Overall deaths/n | Improved deaths/n (%) | Non-improved deaths/n (%) | Fisher *p* |
|---|---|---|---|---|
| T1 ≤1.0 cm | 19/62 | 14/43 (32.6%) | 5/19 (26.3%) | 0.768 |
| T1 >1.0 to 1.3 cm | 37/71 | 11/31 (35.5%) | 26/40 (65.0%) | 0.017 |
| T1 >1.3 to <1.7 cm | 33/94 | 9/27 (33.3%) | 24/67 (35.8%) | 1.000 |
| All | 89/227 | 34/101 (33.7%) | 55/126 (43.7%) | 0.135 |

Two-sided Fisher exact tests compared 60-day mortality between serial TAPSE groups within each baseline stratum. Within-stratum comparisons were exploratory.

## Table S7. TAPSE measures and 60-day mortality

### Panel A. Cox regression

| Exposure and adjustment | Set | HR (95% CI) | *p* |
|---|---|---|---|
| Baseline TAPSE, per 0.1 cm | C | 0.987 (0.918–1.060) | 0.713 |
| Absolute T2 TAPSE, per 0.1 cm | C | 0.960 (0.917–1.006) | 0.086 |
| ΔTAPSE, per 0.1 cm; adjusted for T1 | C | 0.960 (0.916–1.007) | 0.093 |
| ΔTAPSE, per 0.1 cm; adjusted for T2 | C | 0.999 (0.928–1.076) | 0.982 |
| Binary status; adjusted for T1 | C | 1.723 (1.062–2.796) | 0.028 |
| Binary status; adjusted for T2 | C | 1.360 (0.783–2.362) | 0.275 |
| Binary status; adjusted for T1 | F | 1.734 (1.056–2.846) | 0.030 |
| ΔTAPSE, per 0.1 cm; adjusted for T1 | F | 0.960 (0.914–1.008) | 0.099 |
| Absolute T2 TAPSE, per 0.1 cm | F | 0.961 (0.916–1.007) | 0.096 |
| Binary status; adjusted for T2 | F | 1.378 (0.794–2.394) | 0.255 |
| ΔTAPSE, per 0.1 cm; adjusted for T2 | F | 0.995 (0.923–1.073) | 0.897 |

### Panel B. Spline and signed-change analyses

| Comparison | $\chi^2$ | df | *p* |
|---|---|---|---|
| ΔTAPSE spline overall, conditional on T1 | 3.701 | 2 | 0.157 |
| ΔTAPSE spline nonlinearity, conditional on T1 | 0.746 | 1 | 0.388 |
| ΔTAPSE spline overall, conditional on T2 | 0.747 | 2 | 0.688 |
| ΔTAPSE spline nonlinearity, conditional on T2 | 0.746 | 1 | 0.388 |
| T2 TAPSE nonlinearity | 0.106 | 1 | 0.745 |
| Continuous ΔTAPSE added to nonlinear T2 | 0.000 | 1 | 0.996 |
| Signed-change model overall, conditional on T2 | 1.918 | 2 | 0.383 |
| Different slopes below and above ΔTAPSE = 0 | 1.918 | 1 | 0.166 |

### Panel C. Follow-up TAPSE categories

| T2 comparison | HR (95% CI) | Raw *p* | Holm *p* |
|---|---|---|---|
| T2 >1.4 vs T2 ≤1.4 cm | 0.667 (0.427–1.043) | 0.076 | 0.152 |
| T2 >1.7 vs T2 ≤1.7 cm | 0.713 (0.416–1.221) | 0.217 | 0.217 |

All analyses used 227 patients and 89 deaths within 60 days after T2. C and F are defined in Table S4. Models included the adjustment set and TAPSE terms shown; ΔTAPSE = T2 − T1. Binary effects compare Non-improved with Improved patients.

Spline knots were −0.4, 0.2, and 0.9 cm for ΔTAPSE and 0.9, 1.4, and 2.1 cm for T2 TAPSE (10th, 50th, and 90th percentiles). Likelihood-ratio tests assessed overall associations and nonlinearity. The signed-change model included min(ΔTAPSE, 0) and max(ΔTAPSE, 0), each per 0.1 cm, with C and T2 TAPSE.

Panel C adjusted for C, with Holm correction for the two comparisons. Values equal to the cutoff belonged to the lower group. For >1.4 cm, patient/death counts were 101/34 above the cutoff and 126/55 below or equal to it; for >1.7 cm, they were 54/17 and 173/72.

## Table S8. Alternative fixed ΔTAPSE improvement thresholds

| Strict threshold (cm) | Observed rise (cm) | Improved / Non-improved | C + T1 HR (95% CI) | *p* | F + T1 HR (95% CI) | *p* |
|---|---|---|---|---|---|---|
| >0.10 or >0.15 | ≥0.2 | 127 / 100 | 1.613 (1.016–2.560) | 0.043 | 1.653 (1.035–2.639) | 0.035 |
| >0.20 or >0.25 | ≥0.3 | 101 / 126 | 1.723 (1.062–2.796) | 0.028 | 1.734 (1.056–2.846) | 0.030 |
| >0.30 | ≥0.4 | 81 / 146 | 1.714 (1.039–2.827) | 0.035 | 1.693 (1.023–2.803) | 0.041 |

Each model uses 227 patients and 89 deaths and compares Non-improved with Improved patients. The primary threshold is >0.20 cm. At 0.1-cm measurement precision, the five fixed cutoffs produce three distinct groupings. C and F are defined in Table S4.

**Figure S1. Serial TAPSE, estimated PASP, and TAPSE/PASP**

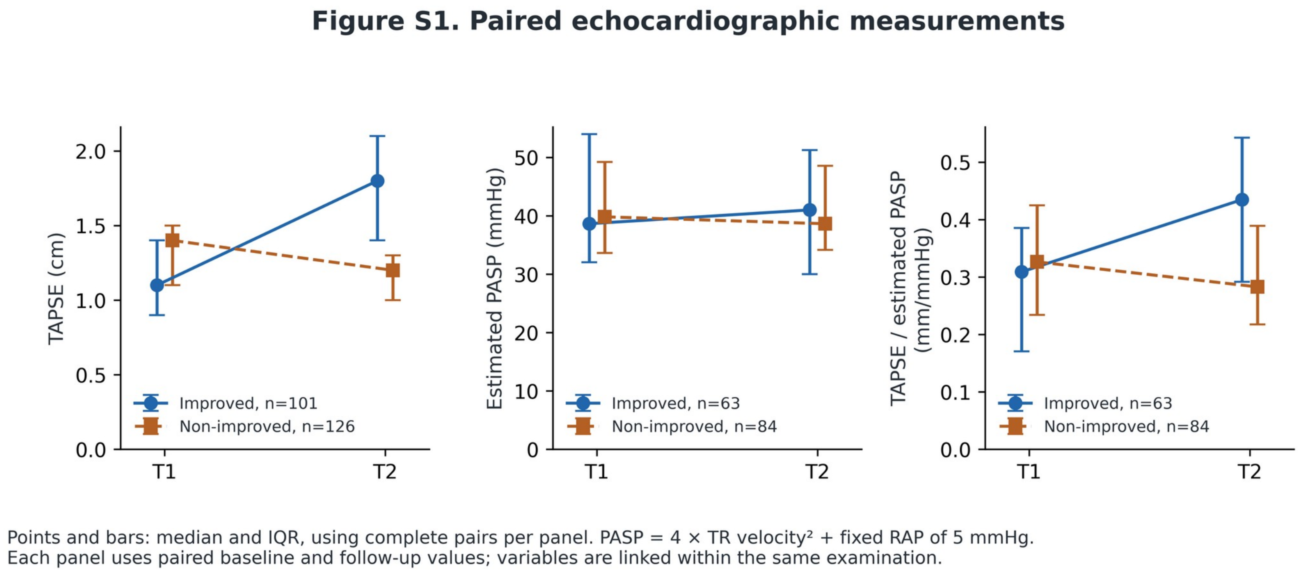


Points and bars show medians and interquartile ranges in complete pairs. TAPSE uses 227 patients (101 Improved and 126 Non-improved). Estimated PASP and TAPSE/PASP use the 147 patients with TR velocity at both selected examinations (63 and 84, respectively). PASP assumes right atrial pressure of 5 mmHg. Comparisons are descriptive because TAPSE is part of both the exposure and the ratio; exact values and tests are in Table S1.

## Figure S2. ΔTAPSE distribution and continuous association

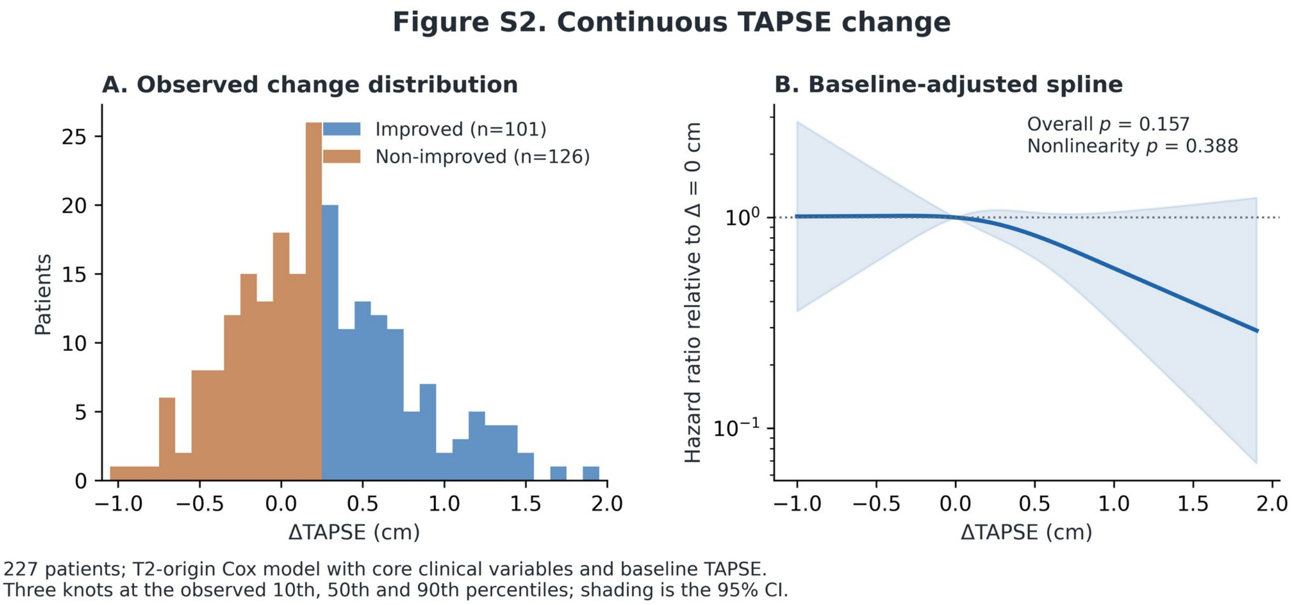


Panel A displays ΔTAPSE in 227 patients. Mean (SD) was 0.239 (0.529) cm; median [IQR] was 0.20 [−0.10 to 0.55] cm; range was −1.0 to 1.9 cm. There were 67 decreases, 18 unchanged measurements, and 142 increases. Panel B shows the T2-origin Cox model with clinical set C, baseline TAPSE, and a three-knot restricted cubic spline for ΔTAPSE. Knots were −0.4, 0.2, and 0.9 cm; the reference is ΔTAPSE = 0. Shading denotes the 95% confidence interval. Overall $p = 0.157$ and nonlinearity $p = 0.388$.

# Figure S3. Follow-up TAPSE and 60-day survival

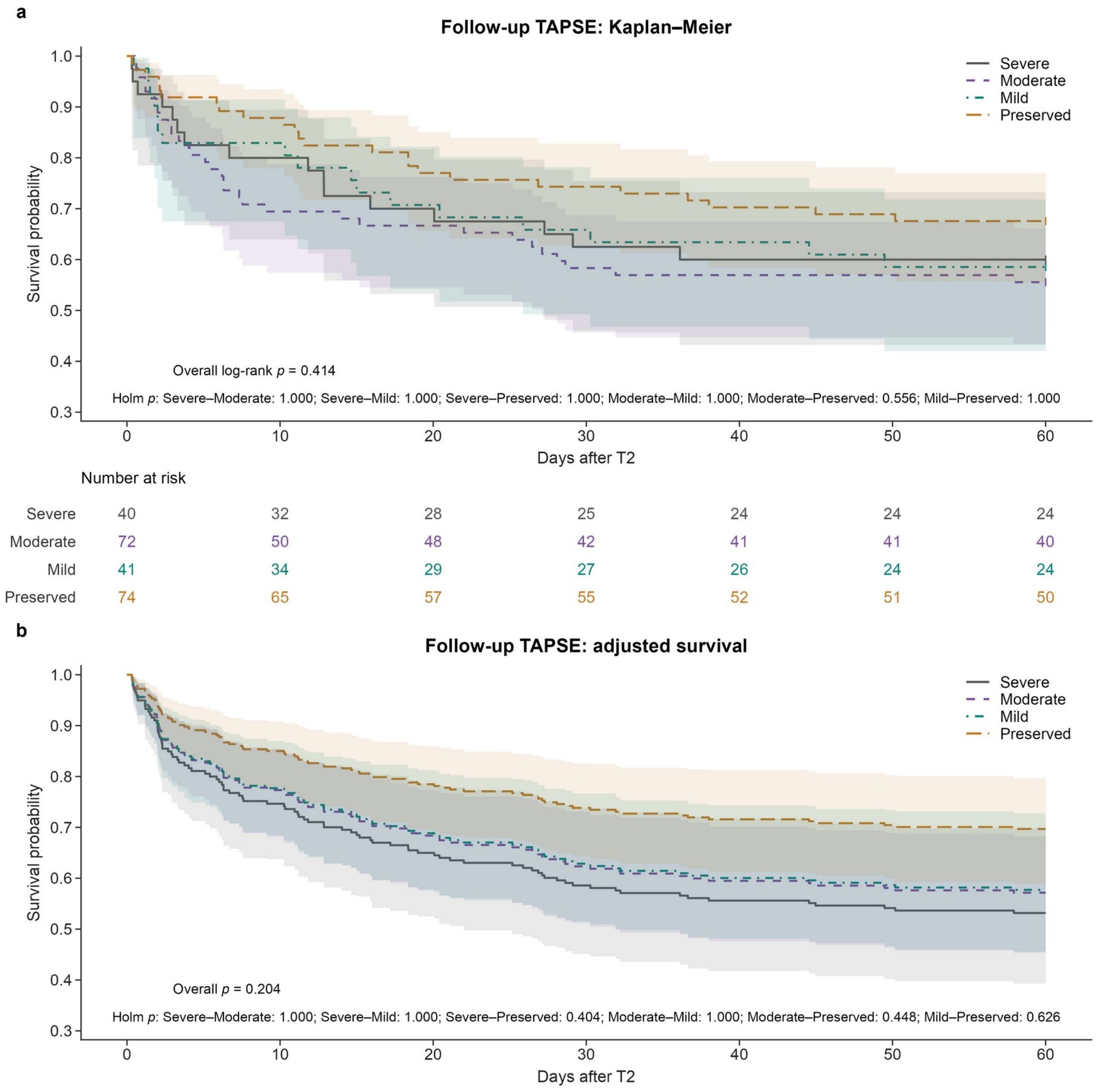


All 227 patients were followed for 60 days after T2. Group labels denote TAPSE ≤1.0 (Severe), >1.0–1.3 (Moderate), >1.3–<1.7 (Mild), and ≥1.7 cm (Preserved). (a) Kaplan–Meier curves. (b) Cox-adjusted survival curves, averaged over the cohort and adjusted for age, sex, Charlson comorbidity index, first-day SOFA score, heart failure, sepsis, COPD, and baseline TAPSE. Shading shows 95% confidence intervals; those in b used 1,000 bootstrap samples. Ticks mark censoring. Overall *p* values use a log-rank test in a and a likelihood-ratio test in b. Pairwise *p* values use log-rank tests in a and Wald contrasts in b, with Holm correction for six comparisons per panel. TAPSE, tricuspid annular plane systolic excursion; SOFA, Sequential Organ Failure Assessment; COPD, chronic obstructive pulmonary disease.
